# Supplementary figures and images for: VMP1 Regulated by chi-miR-124a Effects Goat Myoblast Proliferation, Autophagy, and Apoptosis through the PI3K/ULK1/mTOR Signaling Pathway
Source: Cells. 2022 Jul 18;11(14):2227. doi: 10.3390/cells11142227 (PMC9319091; doi:10.3390/cells11142227)

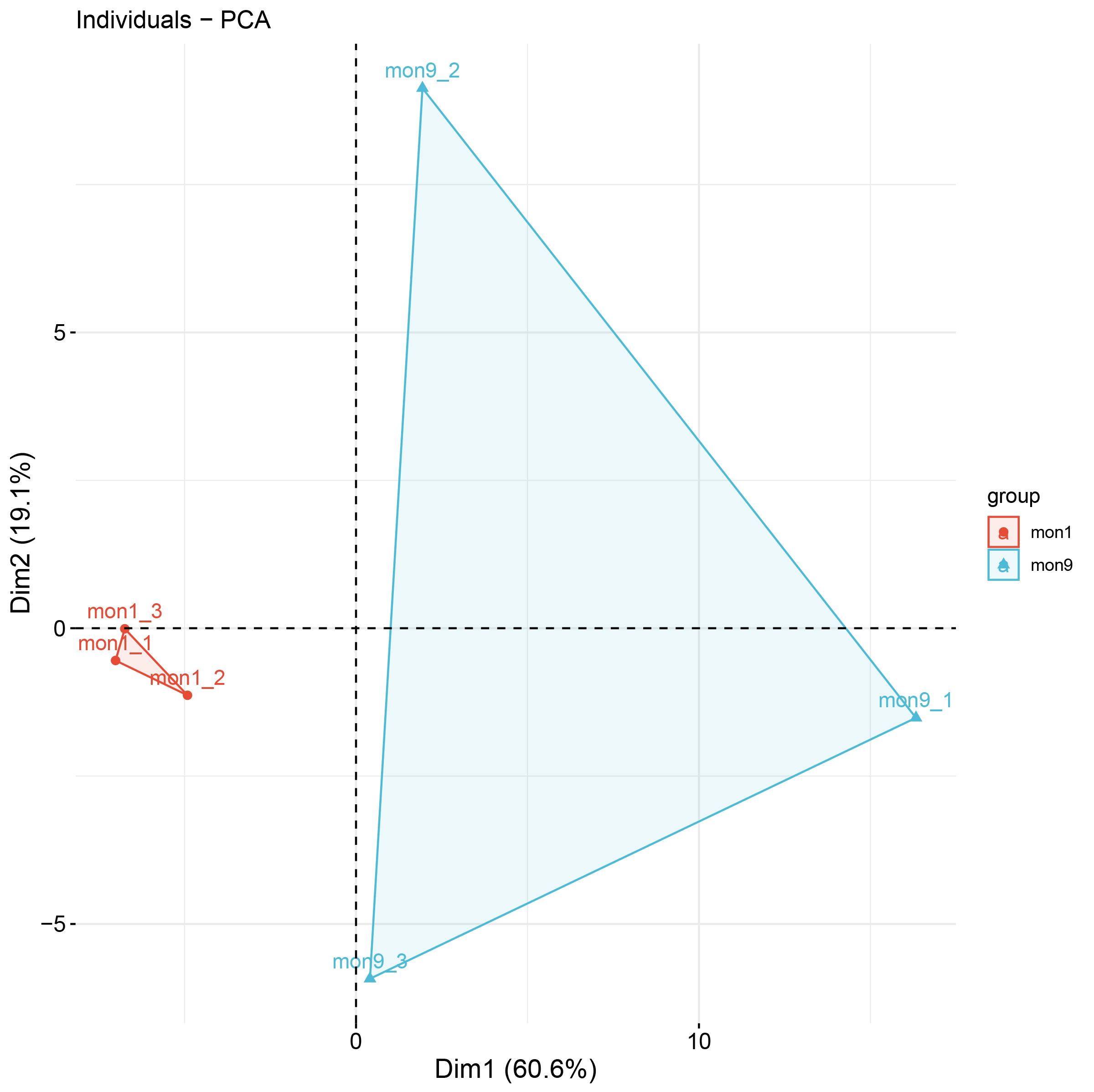

Supplement: Supplementary file 1 [file cells-11-02227-s001.zip › Figure S1.tif]
